# Determining the relative importance of risk and protective factors for adjustment disorder symptoms during the COVID-19 pandemic by mixed-effects random forests

**DOI:** 10.1017/S0033291726104048

**Published:** 2026-06-04

**Authors:** Annett Lotzin, Emily Finne, Georg Schildbach, Elena Acquarini, Dean Ajdukovic, Marina Ajdukovic, Xenia Anastassiou-Hadjicharalambous, Vittoria Ardino, Ida Hensler, Filip K. Arnberg, Maria Böttche, Małgorzata Dragan, Margarida Figueiredo-Braga, Odeta Gelezelyte, Piotr Grajewski, Simon Groen, Marie-José van Hoof, Jana Darejan Javakhishvili, Evaldas Kazlauskas, Chrysanthi Lioupi, Brigitte Lueger-Schuster, Luisa Sales, Lela Tsiskarishvili, Irina Zrnic Novakovic, Ingo Schäfer, ADJUST Study Consortium

**Affiliations:** 1 Department of Psychiatry and Psychotherapy, University Medical Center Hamburg-Eppendorf, Hamburg, Germany; 2 Department of Psychology, Institute for Clinical Psychology and Psychotherapy, MSH Medical School Hamburg, Germany; 3 Institute of Social Medicine and Epidemiology, University of Luebeck, Luebeck, Germany; 4 Institute of Electrical Engineering in Medicine, University of Luebeck, Luebeck, Germany; 5 DISCUI, University of Urbino, Italy; 6 Department of Psychology, Faculty of Humanities and Social Sciences, University of Zagreb, Croatia; 7 Department of Social Work, Faculty of Law, University of Zagreb, Croatia; 8 Psychology Program, School of Humanities, Social Sciences and Law, University of Nicosia, Cyprus; 9 Centre for Psychiatric Research, Department of Clinical Neuroscience, Karolinska Institutet, Stockholm, Sweden; 10 National Centre for Disaster Psychiatry, Department of Medical Sciences, Uppsala University, Uppsala, Sweden; 11 Division of Clinical Psychological Intervention, Freie Universität Berlin, Germany; 12 Trauma Research Laboratory, Faculty of Psychology, University of Warsaw, Poland; 13 Department of Clinical Neurosciences and Mental Health, Faculty of Medicine, University of Porto, Portugal; 14 Trauma Observatory, Centre for Social Studies (CES), University of Coimbra, Portugal; 15 Center for Psychotraumatology, Institute of Psychology, Faculty of Philosophy, Vilnius University, Lithuania; 16 De Evenaar, Centre for Transcultural Psychiatry, GGZ Drenthe, Assen, the Netherlands; 17 iMindU GGZ, Leiden, the Netherlands; 18 Amsterdam UMC, Amsterdam, the Netherlands; 19 Faculty of Arts and Science, Institute of Addiction Studies, Ilia State University, Tbilisi, Georgia; 20 Unit of Psychotraumatology, Department of Clinical and Health Psychology, Faculty of Psychology, University of Vienna, Austria; 21 Unit of Psychiatry, Hospital Militar, Coimbra, Portugal; 22 Faculty of Arts and Science, Ilia State University, Tbilisi, Georgia

**Keywords:** adjustment disorder, community stressors, machine learning, mixed-effects regression random forests, pandemic, random forests

## Abstract

**Background:**

The COVID-19 pandemic exposed individuals to numerous psychosocial and health-related stressors associated with adjustment disorder (AjD) symptoms, yet it remains unclear which factors are most predictive.

**Methods:**

Using mixed-effects regression random forests (MERF), a machine learning approach that combines random forests with mixed-effects regressions, we analyzed longitudinal data from 15,155 adults across 11 European countries collected at three time points between June 2020 and January 2022. We evaluated 245 candidate predictors, including sociodemographic, pandemic-related, and health-related factors, for their relative importance in predicting AjD symptoms (ADNM-8).

**Results:**

The seven most influential predictors, ranked in descending order of importance, were uncertainty about the pandemic’s duration and risks, poor health, social isolation, conflicts at home, loss of daily structure, fear of infection, and restricted personal contact with close others.

**Conclusions:**

AjD symptoms were most strongly linked to factors related to lack of control (e.g., uncertainty, loss of daily structure, fear of infection), as well as current poor health and reduced social connectedness. Interventions that enhance a sense of control through clear communication, help individuals re-establish daily routines, and strengthen social connectedness may mitigate AjD symptoms during future public health crises. Our findings also highlight the potential of machine learning approaches for identifying complex patterns across high-dimensional predictors of clinical symptoms, which may improve prediction accuracy in mental health research.

## Introduction

The COVID-19 pandemic has exposed individuals worldwide to an unprecedented constellation of stressors, ranging from health threats and economic hardship to social isolation and family conflict. Such adverse circumstances are known to increase vulnerability to adjustment disorder (AjD), a diagnosis characterized by persistent preoccupation with stressors and marked impairment across social, occupational, and personal domains (WHO, 2021). Although AjD is particularly relevant in contexts of prolonged and multifaceted stress, it has received far less empirical attention than other disorders such as depression or anxiety. The pandemic thus offers a unique opportunity to advance our understanding of how diverse psychosocial, health-related, and pandemic-specific burdens contribute to AjD symptoms in the general population.

Despite an existing body of research, important knowledge gaps remain. Most existing studies on AjD during the pandemic have been cross-sectional and restricted to a limited set of psychosocial predictors (e.g. Ben-Ezra, Hou, & Goodwin, 2021; Dragan, Grajewski, & Shevlin, 2021; Rossi et al., 2020). Previous studies have associated younger age (Rossi et al., 2020), female gender (Ben-Ezra et al., 2021; Dragan et al., 2021; Rossi et al., 2020), unemployment (Dragan et al., 2021), or job loss (Zelviene, Kazlauskas, & Maercker, 2020) with higher levels of AjD symptoms. General health problems, social isolation, and caregiving problems (Ben-Ezra et al., 2021) were also related to higher AjD levels. These studies have provided valuable initial evidence but cannot capture longitudinal associations.

Fewer longitudinal studies on risk factors for AjD have been conducted during the pandemic. One two-wave study in the general Israeli population identified sex, having a pandemic-related job loss or suspension, and intolerance of uncertainty as predictors for AjD symptom levels (Hamama-Raz et al., 2021). One large pan-European study (ADJUST study; Lotzin et al., 2024) examined risk and protective factors associated with AjD symptoms in over 15,000 individuals from 11 countries across 3 waves (June 2020 to January 2022) using linear mixed-effects models. Higher AjD symptoms were associated with female or diverse gender, older age, more frequent pandemic-related news consumption, prior or current mental disorder, trauma exposure, poorer health, governmental crisis management, fear of infection, social and activity restrictions, work-related problems, and difficult housing conditions. Protective factors included self-employment or retirement, employment in healthcare, and at least weekly face-to-face contact with close others.

The existing longitudinal studies on AjD have relied on linear (mixed-effects) models (Hamama-Raz et al., 2021; Lotzin et al., 2024). While such models may convey valuable insight into significant longitudinal associations between risk and protective factors and AjD symptoms, they are less suited to evaluate the relative importance of a large number of correlated predictors. Linear (mixed) models estimate regression weights to assess associations between predictors and outcomes. Predictors with significant and larger standardized coefficients (*β*) are often seen as more influential, as they correspond to greater differences in the outcome. Regression models offer interpretable coefficients but face limitations when comparing a high number of predictors (Grömping, 2009). Their estimates and corresponding interpretation of individual effects are highly sensitive to the inclusion of other correlated variables in the model due to shared variance. If one of the predictors is removed, the estimates for the others may change. Issues of sensitivity to model specification, multicollinearity, and convergence problems limit the ability of regression-based approaches to robustly determine the relative importance of individual predictors (Grömping, 2009, 2015).

To address these limitations, advanced machine learning methods offer a promising alternative. These methods go beyond the assumptions of classical regression. Random forests are one such approach that can accommodate hundreds of predictors, do not rely on distributional assumptions, capture non-linear associations, and inherently model complex interactions without requiring prior specification (Breiman, 2001; Genuer & Poggi, 2020). Instead of estimating effect sizes or *p*-values, random forests provide a ranking of variable importance. Thus, random forests may provide a robust alternative to conventional linear models when analyzing complex, high-dimensional data, enhancing prediction accuracy in mental health research.

However, standard random forests cannot account for repeated measurements in longitudinal data. Mixed-effects regression random forests (MERF; Hajjem, Bellavance, & Larocque, 2014) overcome this limitation by integrating random effects, thereby combining the predictive power of random forests with the ability to model within-person dependency over time. This approach has rarely been applied in clinical psychology and, to our knowledge, has not yet been used to study AjD in a large-scale, longitudinal, multinational dataset.

The present study fills these gaps by applying MERF to data from more than 15,000 adults across 11 European countries, assessed at three time points between June 2020 and January 2022. Leveraging 245 potential predictors, including sociodemographic, health-related, and pandemic-specific factors, we aim to generate a comprehensive and data-driven ranking of the most important contributors to AjD symptoms. By doing so, this study moves beyond merely identifying significant associations and instead provides a first attempt at identifying a robust hierarchy of predictors, highlighting those factors with the greatest predictive weight. Our findings have the potential to refine theoretical models of AjD, inform targeted screening, and guide the development of focused interventions to mitigate maladaptive adjustment during future public health crises.

## Methods

### Study design and setting

This secondary analysis utilized data from the ADJUST study (Lotzin et al., 2020). The study investigated longitudinal associations between risk and protective factors, stressors, and symptom levels of AjD in the general population across 11 European countries during the COVID-19 pandemic. Data were collected at three time intervals, each 6 months apart, between June 2020 and January 2022 (T1: June 2020 to December 2020; T2: December 2020 to June 2021; T3: June 2021 to January 2022). The ADJUST study was pre-registered in the Open Science Framework (OSF) registry (doi:10.17605/OSF.IO/8XHYG).

### Participants

Participants were recruited from the general populations of 11 European countries: Austria, Croatia, Georgia, Germany, Greece, Italy, Lithuania, the Netherlands, Poland, Portugal, and Sweden. Inclusion criteria were (1) being at least 18 years old, (2) the ability to read and write in the respective national language, and (3) providing informed consent to participate in the study.

### Measures

#### Symptoms of adjustment disorder

AjD symptoms were assessed with the Adjustment Disorder – New Module 8 (ADNM-8; Kazlauskas, Gegieckaite, Eimontas, Zelviene, & Maercker, 2018), an eight-item self-report scale based on ICD-11 criteria. Participants identified the most distressing event during the COVID-19 pandemic and rated how often they experienced related reactions (e.g. ‘I have to think about the stressful situation repeatedly’) on a four-point Likert scale (1 = never to 4 = often). Total scores range from 8 to 32, with scores >22 indicating probable AjD. The ADNM-8 has demonstrated strong structural and convergent validity and high diagnostic accuracy in previous studies (Ben-Ezra, Mahat-Shamir, Lorenz, Lavenda, & Maercker, 2018; Kazlauskas, Zelviene, Lorenz, Quero, & Maercker, 2017).

#### Predictors of AjD symptoms

Risk and protective factors were selected based on the WHO framework for social determinants of health (Solar & Irwin, 2010), adapted to the context of the COVID-19 pandemic (Lotzin et al., 2020). These included sociodemographic, health-related, and pandemic-related characteristics (see Supplement 1 for individual items).

*Sociodemographic characteristics.* We assessed participants’ age, gender (‘female’, ‘male’, ‘diverse’), nationality, and migration or refugee experience (‘yes’, ‘no’). Educational level was assessed using four categories (‘<9 years of schooling’, ‘10–13 years’, ‘completed vocational studies’, ‘completed studies or doctorate’). Household income was assessed as ‘very low’, ‘low’, ‘medium’, or ‘high’. Participants also reported their relationship status (‘single’, ‘temporary relationship’, ‘stable relationship, living separately’, ‘stable relationship, living together’), whether they had children (‘yes’, ‘no’), number of children, and whether they lived alone (‘yes’, ‘no’). Further items assessed living situation, including cohabitation with others (‘yes’, ‘no’; if yes, with how many people), type of cohabitants (‘parents’, ‘partner’, ‘partner and children’, ‘children only’, ‘colleagues/fellow students’, ‘others’), and number of close friends. Work-related variables included current employment status (‘vocational training or study’, ‘part-time employment’, ‘full-time employment’, ‘self-employment’, ‘freelancer’, ‘retired’, ‘seeking work’) and weekly working hours. Pandemic-specific job characteristics covered employment sector (‘health care’, ‘public security’, ‘retail’, ‘services’, ‘maintenance/repair/construction’, ‘education’, ‘other’), whether the work required physical social contact (‘yes’, ‘no’), income loss due to the pandemic (‘yes’, ‘no’) and amount of income reduction, as well as receipt of governmental financial support for pandemic-related financial hardships (‘yes’, ‘no’, ‘not answered’).

*Health-related characteristics.* Participants rated their current general health status (‘very good’, ‘good’, ‘satisfactory’, ‘poor’, ‘very poor’) and indicated whether they had tested positive for the coronavirus (‘yes’, ‘no’). If yes, they reported disease severity (‘mild’, ‘moderate’, ‘severe’), whether they had been hospitalized (‘yes’, ‘no’), and whether they had recovered (‘yes’, ‘no’). Participants were also asked if they personally knew someone who had been infected (‘yes, loved ones’; ‘yes, someone else’; ‘no’) and, if so, how severe the illness was (‘mild’, ‘moderate’, ‘severe’, ‘extremely severe (death)’). We also asked whether they considered themselves at risk for severe or life-threatening COVID-19 symptoms (‘yes’; ‘no’).

Participants assessed whether they had been diagnosed with a mental disorder (‘no diagnosis’; ‘diagnosed but recovered’; ‘diagnosed and currently affected’). We only used the first two categories (‘no diagnosis’, ‘diagnosed but recovered’) as predictors in the analysis to avoid overlap with the dependent variable AjD. Psychological trauma exposure, involving actual or threatened death, serious injury, or sexual violence before or during the COVID-19 pandemic, was assessed using the Criterion A items of PTSD Checklist for DSM-5 (PCL-5; Weathers, Keane, Palmieri, Marx, & Schnurr, 2013). Childhood abuse and neglect were measured using the first five items of the Adverse Childhood Experiences (ACE) questionnaire (Felitti et al., 1998). Participants also reported whether they were currently using any of 10 different mental health support services, including specialist literature or guidebooks, online courses, telephone counseling, online counseling, online psychotherapy, online self-help groups, personal counseling, personal psychotherapy, personal self-help groups, other services, or no service use. For each option, participants responded with ‘yes’ or ‘no’. In addition, they indicated which of these services they would prefer to use when experiencing psychological distress (same response options as above).

*Pandemic-related characteristics.* Participants reported the frequency of pandemic-related news consumption using six categories (from ‘I do not watch, read or listen to news’ to ‘more than 3 hours a day’). They also indicated the frequency of physical and digital social contact with friends and loved ones, each assessed on a four-point scale (from ‘no contact’ to ‘3–6 times a week/every day’). Additionally, participants were asked whether they spent more time at home due to the pandemic (‘no’; ‘yes, as a precautionary measure’; ‘yes, because of own infection’; ‘yes, because of quarantine’), how many weeks they had been spending more time at home, and how many hours per day they currently spent outside their home on average. The overall strictness of lockdown measures was assessed at the individual level using the Oxford COVID-19 Stringency Index (Roser, 2021).

Pandemic-related coping behaviors were measured using the 13-item Pandemic Coping Scale (PCS; Lotzin et al., 2022a). Participants rated the extent to which they engaged in different coping strategies on a 4-point scale (0 = ‘I have not been doing this at all’ to 3 = ‘I’ve been doing this a lot’).

*Pandemic-related stressors.* The burden of pandemic-related stressors was assessed using the 30-item Pandemic Stressor Scale (PaSS; Lotzin et al., 2022b) which covers nine stressor domains: crisis management and communication, fear of infection, burden of infection, restricted personal contact, restricted activity, restricted leisure activity, work-related problems, difficult housing conditions, and restricted access to resources. Participants rated the extent to which they felt burdened by each stressor over the past month on a 4-point scale (0 = ‘not at all burdened’ to 3 = ‘strongly burdened’). Details on item development and psychometric properties are reported elsewhere (PaSS; Lotzin et al., 2022b).

*Pandemic-related positive outcomes.* Participants evaluated 13 items on a 4-point scale (0 = ‘not at all positive’ to 3 = ‘strongly positive’) on the perceived positive consequences of the pandemic across six domains: social cohesion (cohesion in society; more quality time with loved ones, friends, or pet), health (appreciation of own health or health of loved ones; appreciation of good quality of healthcare in own country), work (working from home; new job opportunity or more work orders; (potentially) increased income), learning (learning new skills to solve problems; learning new communication technologies), time (less working hours; more time for enjoyable activities), and re-sorting (time to recover from normal daily stress; time to rethink priorities in life).

*Resilience.* Resilience was measured using the 9-item Resilience Evaluation Scale (RES; Van Der Meer et al., 2018), which focuses on self-confidence and self-efficacy following adversity. Participants rated how they generally feel about themselves and respond to stressful situations on a 5-point scale (0 = ‘completely disagree’ to 4 = ‘completely agree’).

#### Procedure

Risk and protective factors, stressors, and AjD symptoms were assessed at three time intervals, each spaced 6 months apart, between June 2020 and January 2022 using an online survey. Sociodemographic variables were only assessed at baseline. Due to pandemic-related restrictions, recruitment was conducted primarily online. To ensure diversity in gender, age, and education, multiple recruitment strategies were employed. The study was promoted via social media platforms, leisure and interest groups, newsletters, advertisements in newspapers and magazines, as well as through universities, interest groups, and professional associations (for details, see Lotzin et al., 2021). Interested individuals received an invitation link to the online survey. Upon providing informed consent, participants completed the survey on a General Data Protection Regulation (GDPR)-compliant web-based platform.

### Data analysis

#### Data preparation

We included all participants with at least one valid measurement of AjD symptoms at either T1, T2, or T3, resulting in an analysis sample of *N* = 15,155. Participants with missing values on the dependent variable at all three time points (7.7%) were excluded, as we did not impute the dependent variable. Missing values in the independent variables were imputed using a random forest algorithm implemented in the R package *missRanger* (version 2.6.1., Mayer, 2024) employing 100 trees and predictive mean matching (PMM; Stekhoven & Bühlmann, 2012). Simulation studies have shown that the random forest algorithm outperforms multivariate imputation by chained equations (MICE) in both linear and logistic regression models (Stekhoven & Bühlmann, 2012).

The applied imputation method has been shown to be effective for a proportion of missing values of up to 30% (Stekhoven & Bühlmann, 2012), and therefore, 245 predictors assessed in the ADJUST study with less than 30% missing data (except 37% for migration background) were included in the imputation model. For variables assessed at all three time points, all available data were incorporated into the random forest imputation. Variables assessed only at T1 were included once. The latter approach included gender, age, nationality, migration history, education, income, living situation (relationship status, residential situation, having children, having close friends), work situation (employment status, sector, physical contact at work), history of mental illness, adverse childhood experiences, pre-pandemic trauma, and resilience. Individual item responses, rather than summary scores, were used to allow for assessment of the relative importance of each item. To mitigate potential bias introduced by categorical variables with many levels in random forests (see, for instance, Strobl, Boulesteix, Zeileis, & Hothorn, 2007), all categorical variables were dummy-coded, using the most prevalent category as the reference. Continuous variables were standardized using the mean and standard deviation from the baseline (T1) assessment.

Of the 15,155 participants in the dataset, 4,693 (31.0%) provided adjustment disorder (AjD) scores at all three time points. Cases with available scores dropped from 15,088 (99.6%) at T1 to 6,716 (44.3%) at T2 and 5,639 (37.2%) at T3. Predictor missingness ranged from 0.01% to 37.0%.

### Statistical analysis

All analyses were conducted using R version 4.4.2 for Windows (R Core Team, 2023). A mixed-effects regression forest (MERF; Hajjem et al., 2014) based on regression trees was used to predict AjD symptom severity (ADNM-8 total score) across the time points.

A random forest (Breiman, 2001; Genuer & Poggi, 2020) consists of many decision trees, each trained on a bootstrap sample and using a random subset of predictors. Random forests iteratively split the data at each node of a tree by selecting the variable and split point that minimize within-group outcome variance. Starting from the root node (entire sample), this process continues until a stopping criterion – such as minimum node size – is met. The individual predictions of all trees are aggregated to produce a final outcome (Breiman, 2001). This non-parametric method inherently captures interaction effects between predictors, as each split allows for different variables or values to influence subsequent splits. For instance, data may first split by gender and then by age within each group, with variable importance differing between subgroups.

This ensemble approach addresses the limitations of single trees, such as instability and local optimization bias. For continuous outcomes like the ADNM-8 score, performance is measured by minimizing the mean squared error (MSE). Unlike traditional regression, model performance in machine learning is assessed using a training-validation split. Random forests also use out-of-bag (OOB) error estimates, leveraging unused data from each bootstrap sample to internally validate the model. This approach leads to R^2^ values that are better generalizable to new data, while linear regression models estimate R^2^ on the overall training data which may lead to overly optimistic R^2^ values.

MERF combines random forests with linear mixed-effects modeling, capturing both fixed effects (via random forest) and random effects to account for intra-individual correlations over time (Hu & Szymczak, 2023). It accommodates longitudinal data by using random forests to model complex fixed effects while simultaneously estimating random effects to reflect dependencies from repeated measurements. The estimated model can be specified as follows (Hu & Szymczak, 2023):

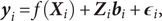




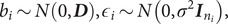

where **y**_i_ is the predicted outcome vector of individual i comprising the n_i_ observations of outcome y for this individual, **X**_i_ refers to the matrix containing the fixed-effects predictors, **Z**_i_ is the matrix with random-effects predictors, **b**_i_ is the vector with the random-effects coefficients, and **ϵ**_i_ includes the random errors. As in a standard mixed-effects model, both **b**_i_ and **ϵ**_i_ are assumed to follow a normal distribution with mean 0 and covariances **D** and σ^2^**I**_ni_.

Unlike conventional linear mixed models that assume linearity in predictors, MERF estimates the fixed-effects function f(**X**_i_) using random forests. An expectation–maximization (EM) algorithm iteratively alternates between fitting the forest (adjusted for random effects) and updating the random effects based on the forest’s predictions, until convergence. The MERF algorithm has demonstrated notable efficacy in handling high-dimensional longitudinal data, as evidenced by a simulation study (Capitaine, Genuer, & Thiébaut, 2021).

We applied the MERFranger function from the SAEforest R package (version 1.0.1, Krennmair & Frink, 2023). This function integrates the ranger package for random forests and lme4 for mixed-effects modeling. MERFranger was chosen over alternatives (REEMforest, MERF in LongituRF) due to comparable accuracy and substantially faster computation on a subset of our data (Probst, Wright, & Boulesteix, 2019).

The model included all variables and time points (T1-T3; T1 as reference) as fixed effects. After dummy-coding, 245 predictors (150 binary, 95 continuous) were entered. A random intercept accounted for repeated measures. Data were split into 75% training (20,523 observations) and 25% validation (6,920). The forest used 1,000 trees, with *mtry* = 80, minimal node size of three, and a sample fraction of 0.876 per tree as bootstrap sample. These hyperparameters were selected as the most appropriate according to tuneRanger (version 0.7, Probst et al., 2019).

MERF model performance was evaluated using R^2^ (training/test), out-of-bag (OOB) error, training error, and test error. All errors were quantified as mean squared error (MSE). OOB error reflects the average MSE for observations excluded from bootstrap samples. Training error is the MSE between observed and predicted outcomes in the training set. Test error is the MSE in the validation set, based solely on the fixed-effects (random forest) component, as random effects are estimated only during training. OOB errors and test errors inform about the generalizability of the model to new data which may be overestimated by the training error since it relates to the accuracy of predictions in the known data (Genuer & Poggi, 2020).

#### Relative importance of predictors

Identifying the most important predictors was the primary goal of our analysis. We used the MERFranger function to estimate the relative permutation importance of each of the 245 predictors and their interactions with each other. The MERFranger function ranks predictors by their importance in predicting the outcome. For each variable, values are permuted and the resulting change in out-of-bag (OOB) error is compared to the original model. A larger increase in OOB error indicates higher importance, whereas uninformative variables yield little or no change. Variables are ranked by their predictive importance. We identified those with the highest importance scores by visually examining the graph of the importance trajectory, as there is no established cut-off for defining the most important predictors besides the criterion used in the Boruta algorithm. To assess the robustness of the relative importance values from MERFranger, a sensitivity analysis with the Boruta algorithm using the Boruta package (version 8.0.0) was conducted (Kursa & Rudnicki, 2010). Boruta compares the importance of the predictors to that of randomly permuted, uninformative ‘shadow’ variables across multiple runs (100). Boruta does not consider the random effects but generates a random distribution of importance values and tests these against insignificant random values. Predictors scoring significantly higher than all shadow variables are classified as ‘important’. ‘Unimportant’ variables (i.e. scoring not significantly higher than shadow variables) are iteratively removed.

To obtain effect sizes of the most important predictors derived from the MERF model, we conducted a linear mixed-effects model on the most important predictors afterward (package lme4 v 1.1-37, Bates, Mächler, Bolker, & Walker, 2015). Standardized coefficients from the mixed-effects model were compared descriptively with variable importance estimates derived from a restricted MERF model containing the same predictors to examine convergence between linear effect sizes and machine-learning-based predictive relevance.

#### Interaction strength

Random forests inherently model interactions between predictors. To quantify interaction strength, we used Friedman’s H statistic (Friedman & Popescu, 2008) via the *iml* package (version 0.11.4, Molnar, 2018). H measures how much better a model predicts the outcome when two variables are used together compared to when used separately, ranging from 0 (no interaction) to 1 (strong interaction). For each variable, we computed the average pairwise interaction strength with all others and rescaled H values to a 0–100 scale for readability.

#### Representative tree of the random forest

The order of variable importance and interaction strength alone does not explain how the random forest makes predictions. To overcome this disadvantage of machine learning approaches compared to individual decision trees and to improve interpretability, we generated an artificial tree (ART) based on the most important variables using the R package *timbR* (version 3.0) that summarizes the forest (Kronziel & Laabs, 2025; Laabs, Kronziel, König, & Szymczak, 2024). This newly developed approach creates a single tree from the entire forest that best represents the random forest model in terms of the smallest dissimilarity (pairwise distance) measure, offering a visual and interpretable approximation of the full model. To understand the principle of a decision tree an thus of an ART, imagine a marble run (Figure 2). The marble starts at the top level of the tree and follows a path that branches off at each decision point. At every split, it takes one direction based on a specific rule, continuing downward until it reaches the bottom level – the predicted outcome (in our case the AjD score). To improve interpretability, each variable of the ART was split at the median only.

## Results

### Sample characteristics

Two-thirds of participants were female and more than half had completed a university degree (Table 1). Over half reported medium to high household income. Adjustment disorder (AjD) symptoms showed only minor changes over time, with mean ADNM-8 scores of *M* = 16.0 (*SD* = 6.45) at T1, *M* = 16.5 (*SD* = 6.65) at T2, and *M* = 15.4 (*SD* = 6.47) at T3.

**Table 1.** Sample characteristics (*N* = 15,155)

Variable	Total (*N* = 15,155)		At-risk for adjustment disorder^a^ (*N* = 3,942)		Not at-risk for adjustment disorder (*N* = 11,213)	
	*M*	*SD*	*M*	*SD*	*M*	*SD*
Age	42.7	15.2	43.0	15.1	42.6	15.3
Sex	*n*	*%*	*n*	*%*	*n*	*%*
Male	4,707	31.1	994	25.2	3,713	33.1
Female	10,393	68.6	2,926	74.2	7,467	66.6
Other	54	0.4	22	0.6	32	0.3
Education						
Less than 10 years of school	368	2.4	98	2.5	270	2.4
**≥**10 years of schooling	3,461	22.8	948	24.0	2,513	22.4
Vocational studies	2,141	14.1	641	16.3	1,500	13.4
Completed studies	8,879	58.6	2,213	56.1	6,666	59.4
Missing	306	2.0	42	1.1	264	2.4
Income^b^						
Very low	1,207	8.0	389	9.9	818	7.3
Low	2,706	17.9	756	19.2	1,950	17.4
Medium	5,100	33.7	1,393	35.3	3,707	33.1
High	5,158	34.0	1,169	29.7	3,989	35.6
Missing	984	6.5	235	6.0	749	6.7
Migration background						
Yes	8,223	54.3	2,036	51.6	6,187	55.2
No	1,499	9.9	446	11.3	1053	9.4
Missing	5,433	35.8	1,460	37.0	3,973	35.4
Employment sector						
Healthcare	1,910	12.6	430	10.9	1,480	13.2
Public security	749	4.9	105	2.7	644	5.7
Retail, services	856	5.6	262	6.6	594	5.3
Maintenance, construction	420	2.8	114	2.9	306	2.7
Education	1,795	11.8	459	11.6	1,336	11.9
Not working	2,536	16.7	768	19.5	1,768	15.8
Other	4,788	31.6	1,261	32.0	3,527	31.5
Missing	2,101	13.9	543	13.8	1558	13.9
COVID–19 infection^c^	*n*	*%*	*n*	*%*	*n*	*%*
T1						
Yes	296	2.0	95	2.4	201	1.8
No	14,842	97.9	3,844	97.5	10,998	98.1
Missing	17	0.1	3	0.1	14	0.1
T2						
Yes	632	4.2	195	5.0	437	3.9
No	6324	41.7	2108	53.5	4216	37.6
Missing	8199	54.1	1639	41.6	6560	58.5
T3						
Yes	752	5.0	237	6.0	515	4.6
No	5082	33.5	1655	42.0	3427	30.6
Missing	9321	61.5	2050	52.0	7271	64.9
Lockdown stringency	*M*	*SD*	*M*	*SD*	*M*	*SD*
T1^d^	56.9	9.3	57.2	9.0	56.8	9.4
T2^e^	72.2	8.7	73.4	7.6	71.5	9.1
T3^f^	55.6	10.2	56.1	9.6	55.3	10.4
Adjustment disorder (ADNM–8)						
T1^g^	16.0	6.5	23.5	5.8	13.4	4.2
T2^h^	16.5	6.7	22.7	6.3	13.4	4.2
T3^i^	15.4	6.5	21.4	6.4	12.5	4.0

*Note*: Percentages (%) refer to the numbers of valid cases per category. An ADNM-8-score ≥ 23 indicates at-risk for adjustment disorder.

a Participants were coded as at-risk for adjustment disorder if they exceeded the ADNM-8 cut-off score in at least one wave (T1, T2, or T3).

b Income categories were adapted for each country.

c Numbers of participants currently infected with COVID-19.

d *n* = 15,155.

e *n* = 7,057.

f *n* = 5,716.

g *n* = 15,088.

h *n* = 6,716.

i *n* = 5,639.

COVID-19 infections rose across waves: 296 participants (2.0%) reported an infection at T1, increasing to 632 (4.2%) at T2 and 752 (5.0%) at T3. Lockdown stringency peaked at T2 during winter (*M* = 72.2, *SD* = 8.7), compared to lower levels at T1 (*M* = 56.9, *SD* = 9.3) and T3 (*M* = 55.6, *SD* = 10.2).

Women had a higher risk of AjD (28.1%) compared to men (21.1%). Unemployed individuals were more likely to be at risk, whereas those employed in healthcare or public security were less likely. No consistent association was found between COVID-19 infection and AjD risk.

### Relative importance of predictors

The MERF model, based on 245 predictors, explained *R^2^* = 0.53 of variance in the training data. The OOB error was *MSE* = 12.46, and the training error was *MSE* = 2.32. Including random effects reduced the OOB error compared to the test error and shifted it toward the training error, indicating good generalizability. The person-level random effect had standard deviation of 2.91, and the residual error had a standard deviation of 4.078. In the validation set (25% of cases), the model explained *R^2^* = 0.40 with a test error of *MSE* = 25.86, reflecting the fixed-effect-only predictions.

**Figure 1.**
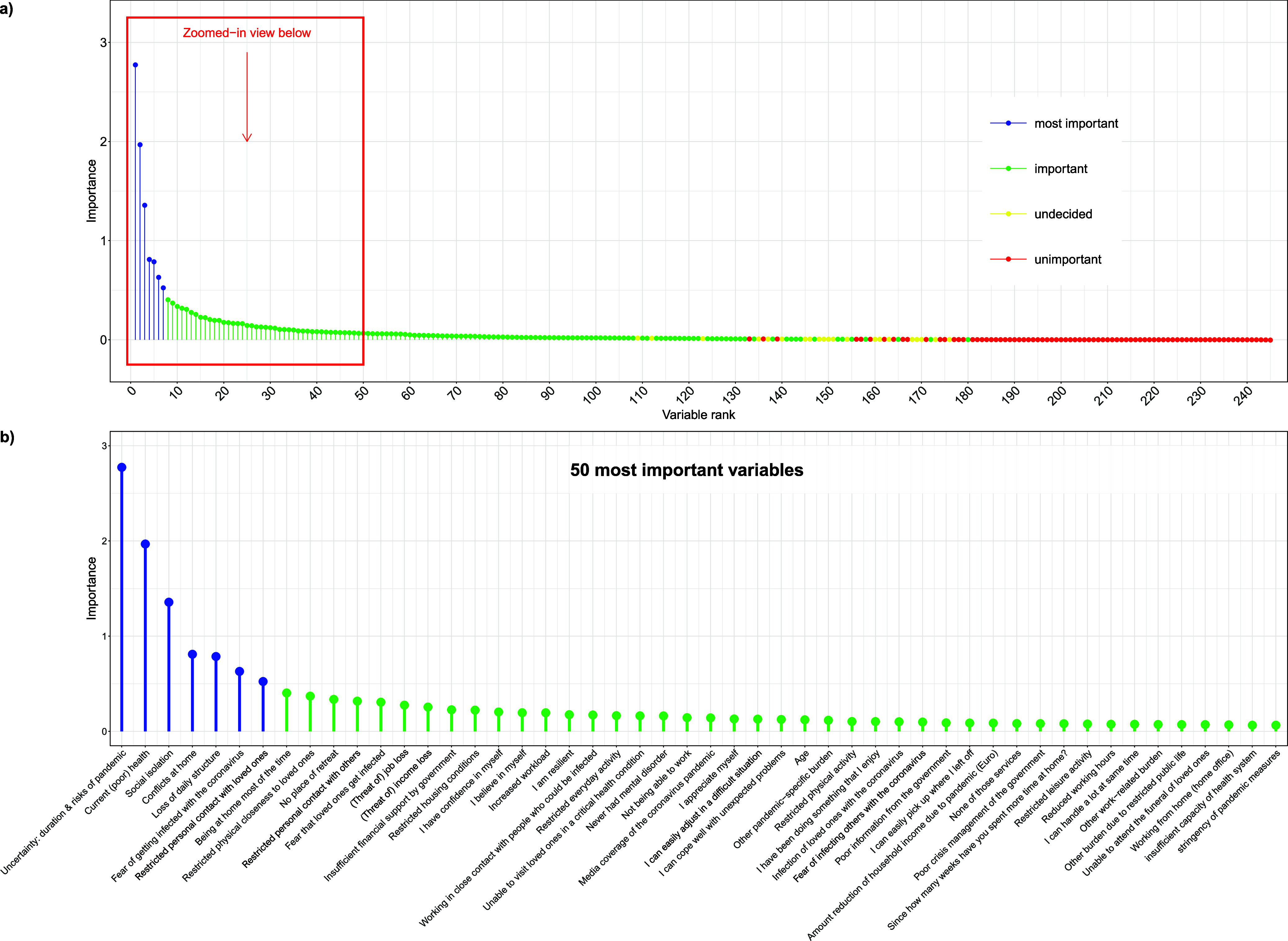
Ranking of Predictor Importance for Adjustment Disorder Symptoms. *Note:* Predictor importance values ranked in descending order, with an enlarged view of the top 50 predictors presented below. Colors indicate importance classifications (“most important”, “important”, “undecided”, “unimportant”). a) Distribution of the permutation importance values of all predictors in the MERF model ranked in a descending order. b) Enlarged cut-out of the first 50 most important variables with name.

The importance curve of the 245 predictors visually showed a drop after the top three predictors, followed by another small drop after the seventh most important variable (Figure 1).

The top seven predictors with particularly high importance values (ranging from 2.78 to 0.52) for predicting adjustment disorder (AjD) symptoms (Table 2) included:

1. Uncertainty about the duration and risks of the pandemic
2. Current poor health
3. Social isolation
4. Conflicts at home
5. Loss of daily structure
6. Fear of getting infected with the coronavirus
7. Restricted personal contact with loved ones

**Table 2.** Association, importance, and interaction strength of the 50 most important predictors for adjustment disorder symptoms

No.	Domain	Item	Associationwith AjD^a^	Importance	Interaction strength
1	Fear of infection	Uncertainty about duration and risks of the pandemic	+	2.773	16.008
2	Current situation	Current (poor) health	+	1.968	13.181
3	Restricted personal contact	Social isolation	+	1.357	17.764
4	Difficult housing condition	Conflicts at home	+	0.810	12.497
5	Restricted activity	Loss of daily structure	+	0.786	9.921
6	Fear of infection	Fear of getting infected with the coronavirus	+	0.630	6.120
7	Restricted personal contact	Restricted personal contact with loved ones	+	0.523	4.486
8	Restricted activity	Being at home most of the time	+	0.403	5.016
9	Restricted personal contact	Restricted physical closeness with loved ones	+	0.370	5.711
10	Difficult housing condition	No place of retreat	+	0.336	3.075
11	Restricted personal contact	Restricted personal contact with others	+	0.318	3.414
12	Fear of infection	Fear that loved ones get infected with the coronavirus	+	0.307	4.042
13	Work-related problems	(Threat of) job loss	+	0.276	4.889
14	Work-related problems	(Threat of) income loss	+	0.255	3.421
15	Work-related problems	Insufficient financial support by government	+	0.227	6.118
16	Difficult housing condition	Restricted housing conditions (little space)	+	0.223	2.279
17	Resilience (T1)	I have confidence in myself	−	0.204	3.396
18	Resilience (T1)	I believe in myself	−	0.196	2.933
19	Work-related problems	Increased workload	+	0.195	2.681
20	Resilience (T1)	I am resilient	−	0.175	3.192
21	Work-related problems	Working in close contact with people who could be infected	+	0.172	1.559
22	Restricted activity	Restricted everyday activity (e.g. shopping)	+	0.165	4.003
23	Restricted face-to-face contact	Unable to visit loved ones in a critical health condition	+	0.164	1.394
24	Previous diagnosis mental disorder	Never had mental disorder	−	0.162	2.013
25	Work-related problems	Not being able to work	+	0.144	2.351
26	Crisis management and communication	Media coverage of the coronavirus pandemic	+	0.142	3.726
27	Resilience (T1)	I appreciate myself	−	0.130	1.919
28	Resilience (T1)	I can easily adjust in a difficult situation	−	0.129	2.444
29	Resilience (T1)	I can cope well with unexpected problems	−	0.125	1.940
30	Socio-demographics	Age	−	0.122	1.803
31		Other pandemic-specific burden	+	0.117	1.188
32	Restricted activity	Restricted physical activity	+	0.104	1.188
33	Joyful Activities	I have been doing something that I enjoy	−	0.103	1.329
34	Burden of infection	Infection of loved ones with the coronavirus	+	0.101	0.956
35	Fear of infection	Fear of infecting others with the coronavirus	+	0.098	2.376
36	Crisis management and communication	Poor information from the government	+	0.090	1.075
37	Resilience (T1)	I can easily pick up where I left off	−	0.088	1.113
38	Current situation	Amount of reduction in household income due to pandemic (Euro)	+	0.087	1.311
39	Current service use	None of those services	−	0.082	1.185
40	Crisis management and communication	Poor crisis management of the government	+	0.082	1.497
41	Current situation	Weeks spent more time at home	+	0.081	2.011
42	Restricted activity	Restricted leisure activity (e.g. restaurant visits)	+	0.077	0.879
43	Work-related problems	Reduced working hours/fewer work orders	+	0.075	1.336
44	Resilience (T1)	I can handle a lot at the same time	−	0.075	2.375
45	Work-related problems	Other work-related burden	+	0.072	1.152
46	Restricted activity	Other burden due to restricted public life	+	0.071	0.716
47	Restricted personal contact	Unable to attend the funeral of loved ones	+	0.071	1.605
48	Work-related problems	Working from home (home office)	+	0.069	0.833
49	Restricted access to resources	Insufficient capacity of the health care system for seriously ill people	+	0.065	1.266
50	Lockdown stringency	Stringency of pandemic measures	+	0.065	2.204

*Note*: Content domains, variable names, importance values, and the direction of association with adjustment disorder symptoms for the 50 most important variables. Importance was assessed with the MERFranger function (permutation method). For interaction strength, Friedman’s H was multiplied by 100 (range 0–100). PCS: Pandemic Coping Scale. Resilience: Resilience Evaluation Scale.

a Positive (+) or negative (−) direction of the unadjusted (bivariate) association with adjustment disorder symptoms.

The remaining variables showed a gradual decline in importance, with no clear drop between more and less relevant predictors. In total, 143 predictors were classified as important, indicating a non-random contribution to the prediction of AjD symptoms. Most of the highest-ranked predictors reflected pandemic-specific stressors rather than general risk factors. All top-ranked variables were positively associated with higher symptom levels, indicating that they functioned as risk factors rather than protective factors. Protective factors such as resilience appeared later in the importance ranking. Stable, pandemic-unrelated factors such as sociodemographic variables – except for age, which ranked 30^th^ – were of relatively low predictive importance. The time of measurement (T2 or T3 vs. T1) also showed low predictive importance.

A random forest (MERF) model using only the seven most important predictors achieved an OOB explained variance of *R^2^* = *0.51* and *MSE* = *12.14* in the training data and *R^2^* = *0.33* with a test error of *MSE* = *28.30* in the validation set. This indicates that these top seven variables, including their interactions, explained a substantial portion of the variance in AjD symptoms and fit the training data nearly as well as the full model.

The predictors with the highest interaction strength (Table 2) were identical to those with the highest overall importance. Specifically, the five top-ranked variables – uncertainty about the duration and risks of the pandemic, current poor health condition, social isolation, conflicts at home, and loss of daily structure – exhibited stronger interaction effects than all other predictors.

The sensitivity analysis of variable importance using the Boruta algorithm closely aligned with the results of the MERF model. Of the 30 most important variables identified by MERF, 28 were also among the top predictors in the Boruta analysis; except age and one resilience item (‘I cope well with unexpected problems’) which were ranked less important. Identical ranking positions were found for 7 of the top 30 variables across both methods, and in six of these 7 cases, the particularly high importance of the leading predictors was confirmed.

A mixed-effects linear regression model incorporating these seven predictors yielded a model fit that was almost as good as the model fit of a reduced MERF (see Table 3). The effect sizes varied between *β* = 0.30 for current poor health and *β* = 0.09 for restricted personal contacts with loved ones. The linear mixed-effects model with these seven predictors achieved a very good model fit, even without including interactions or non-linear relations which was similar to the model fit of the MERF including the same seven predictors.

**Table 3.** Effect estimates of the mixed-effects regression and MERF including the seven most important predictors

Predictor	Unstandardized *b* from mixed-model	*t*-value from mixed-model	Standardized *β* (95% CI) mixed-model	MERF importance^a^
Uncertainty	1.00	22.92	0.23 (0.21, 0.25)	2.77 (31.34)
Current poor health	1.33	34.17	0.30 (0.29, 0.32)	1.97 (22.24)
Social isolation	0.71	13.24	0.16 (0.14, 0.18)	1.36 (15.34)
Conflicts at home	0.92	22.66	0.20 (0.19, 0.22)	0.81 (9.16)
Loss of structure	0.77	16.44	0.17 (0.15, 0.19)	0.79 (8.88)
Fear of infection	0.55	12.75	0.12 (0.11, 0.14)	0.63 (7.12)
Restricted personal contact	0.38	7.52	0.09 (0.06, 0.11)	0.52 (5.91)
Marginal *R* ^2^			0.31	0.34
Conditional *R* ^2^			0.58	0.65
Training error (*MSE*)			12.36	10.39
Test error (*MSE*)			28.57	28.27

*Note*: Marginal *R^2^* includes only the fixed-effects component based on the training data. Conditional *R^2^* additionally includes random effects. In both models, test and training error have the same meaning: the mean of the squared deviations between the model-predicted and observed values. MSE = mean squared error.

a For better interpretation of the relative importance, importance values were scaled to sum up to 100% over the seven predictors).

#### Most representative artificial tree

**Figure 2.**
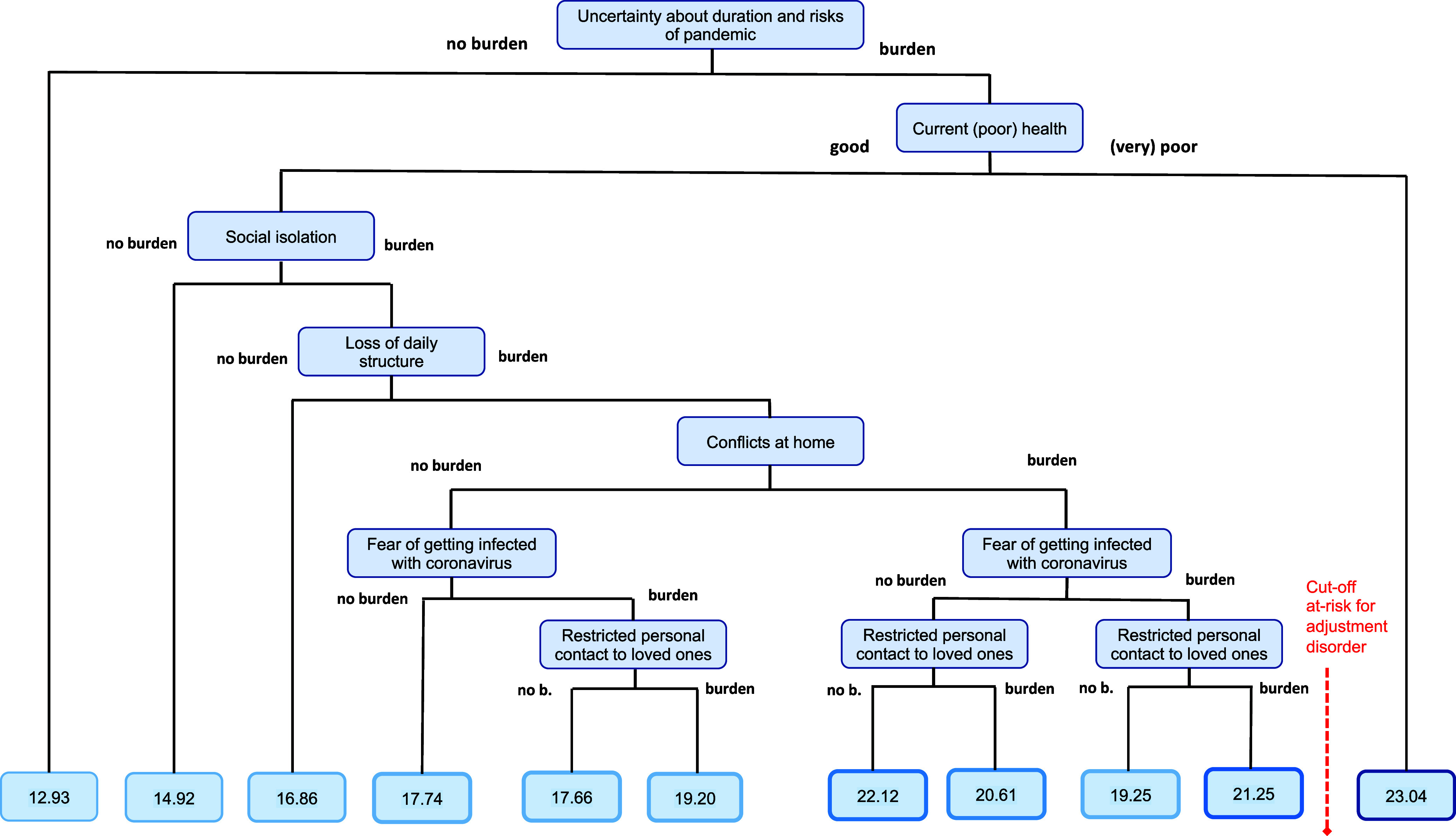
Most Representative Artificial Tree (ART) From the Random Forest. *Note:* Pandemic stressors were dichotomized into ‘no burden’ (not at all or somewhat burdened) vs. ‘burden’ (moderately or strongly burdened). Subjective health was dichotomized into ‘good’ (very good, good or satisfactory) versus ‘(very) poor’ (poor or very poor). ADNM-8 scores range from 8 to 32, with scores ≥ 23 indicating at-risk for adjustment disorder.

The most representative artificial tree (ART) from our random forest, limited to the seven most important predictors, is shown in Figure 2.

The first split at the top is based on the uncertainty about duration and risks of the pandemic. Individuals with low uncertainty about duration and risks of the pandemic (score 0 or 1) had the lowest predicted AjD score (12.9). Those with high uncertainty *and ‘*Current poor health’ had the highest predicted score (>23), exceeding the clinical threshold. Individuals with high uncertainty about the duration and risks *but* good current health showed elevated AjD scores (~22.1) below the cut-off for probable AjD. The AjD scores were higher when participants with good current health reported burden from social isolation, loss of daily structure, conflicts at home, fear of infection, and restricted personal contact with loved ones. The AjD scores of the participants with good current health were lower when they also reported less burden by fear of infection and less reduced contact with loved ones.

## Discussion

In this large longitudinal pan-European study of more than 15,000 adults from the general population, we applied mixed-effects regression random forests (MERF) to identify the most important sociodemographic, pandemic-, and health-related predictors of AjD symptoms during the COVID-19 pandemic. We extend previous research using traditional (mixed) regression analyses by quantifying the relative importance of a broad array of risk and protective factors using MERF, a novel data-driven machine learning approach combining random forests with random-effects analysis.

Our analysis revealed that out of 245 potential predictors of AjD symptoms, the seven most important were uncertainty about the pandemic’s duration and risks, perceived current poor health, social isolation, conflicts at home, loss of daily structure, fear of infection, and restricted personal contact to loved ones. The present findings suggest that some of the most important predictors identified, particularly uncertainty about the pandemic’s duration and risks, fear of infection, and loss of daily structure, may converge around a shared underlying mechanism: perceived lack of control. According to Lazarus and Folkman’s transactional model of stress (Lazarus & Folkman, 1984), psychological distress arises from the appraisal of a situation as threatening in combination with limited perceived control to cope with the stressor. From a behavioral perspective, such a reduced environmental control and disrupted routines may limit opportunities for adaptive coping behaviors, reinforcing avoidance, withdrawal, and rumination (Lewinsohn, 1974). Lack of perceived control is known to intensify stress responses (Maier & Seligman, 2016) and may contribute to the persistence of adjustment disorder symptoms.

Uncertainty about the pandemic’s duration and risks was the risk factor with the highest importance that underscores the central role of uncontrollable, ambiguous stressors for AjD symptoms. Uncertainty has long been recognized as central in conceptualizations of maladaptive stress responses (Peters, McEwen, & Friston, 2017) as it increases threat appraisal and reduces perceived control (Lazarus & Folkman, 1984). Theoretical stress models further posit that uncertainty exacerbates rumination and worry (Carleton, 2016) which are core features of AjD (WHO, 2021).

Fear of infection also emerged as a highly important predictor of AjD symptoms. Fear of infection reflects a direct health-related threat and may intensify primary threat appraisal (Carleton, 2016; Lazarus & Folkman, 1984) particularly when combined with uncertainty regarding disease severity, duration, or long-term consequences. In the context of AjD, persistent infection-related fear may contribute to sustained stressor-related rumination and hinder adaptive coping to changing circumstances.

Loss of daily structure, such as disrupted sleep, work, and social routines, also appeared as a key predictor of AjD symptoms. Daily structure provides stability, self-regulation, and a sense of control; its loss may increase emotional dysregulation and helplessness and may thereby contribute to AjD. Reestablishing structure might thus be a core element in prevention and intervention strategies.

A perceived current poor health condition was an additional important predictor of AjD symptoms. The prominence of a poor health condition mirrors previous mixed-effects analyses in this cohort, where it was the single strongest predictor (Lotzin et al., 2024). Moreover, poor perceived health appeared to amplify the psychological impact of uncertainty about the pandemic, suggesting that individuals who felt physically vulnerable were particularly prone to maladaptive stress responses under conditions of prolonged uncertainty (illustrated in Figure 2).

In accordance with preceding cross-sectional research (e.g. Ben-Ezra et al., 2021; Dragan et al., 2021), social factors such as social isolation and restricted personal contact with loved ones were among the variables with the highest importance for predicting AjD symptoms in the present study. This finding may reflect the psychosocial costs of physical distancing measures. Social support has been identified as a pivotal protective factor in stress adaptation, serving to mitigate the impact of adverse events (Cohen & Wills, 1985). Conversely, social isolation has been demonstrated to deplete psychosocial resources and augment vulnerability to stress (Hobfoll, 1989). From the perspective of stress and coping (Lazarus & Folkman, 1984), the provision of social support has been shown to enhance perceived control and facilitate adaptive emotion regulation. Conversely, a reduction in social support has been demonstrated to intensify perceived uncontrollability and uncertainty, which are core mechanisms in AjD (Maercker et al., 2013).

Conflicts at home are a social stressor that has received comparatively little attention in the field of mental health research. Nonetheless, it has emerged as having a high importance for AjD symptoms in our study, thus underscoring the pivotal role of family environments in facilitating adjustment during continued periods of crisis. This finding suggests that not only the quantity but also the quality of social contacts are significant factors. Social contacts characterized by conflict may exacerbate rather than alleviate AjD symptoms. In accordance with this supposition, preceding studies have documented associations between familial conflict and psychological distress, including symptoms of anxiety and depression, during the course of the pandemic (Lobo et al., 2025).

The present findings extend the existing research on sociodemographic predictors of mental health symptoms by identifying those factors with the highest importance relative to other pandemic- and health-related predictors. While earlier research, predominantly cross-sectional in nature, had identified significant associations between younger age, female gender, and unemployment and elevated AjD symptoms (Rossi et al., 2020; Zelviene et al., 2020), our MERF revealed that these factors carried minimal predictive importance when capturing a wide array of novel risks and stressors introduced by the pandemic. In the present analysis, age emerged as the sociodemographic predictor with the highest importance relative to other sociodemographic predictors, yet it occupied only the 30th position among all predictors (see Figure 1). In contrast to the findings of earlier research which identified significant associations between female gender (Ben-Ezra et al., 2021; Dragan et al., 2021; Rossi et al., 2020) and AjD symptoms, the present study demonstrates that female gender is of relatively low importance as a predictor of AjD symptoms. While women demonstrated a higher severity of AjD symptoms than men also in our study, this gender disparity could be attributed to the influence of pandemic-related stressors, which have been observed to be more prevalent among women. This finding may suggest that studies which only include a limited number of (predominantly sociodemographic) predictors may overestimate the importance of sociodemographic variables on AjD symptoms.

In contrast to sociodemographic predictors, stressors specific to the pandemic emerged as relatively more significant factors within a comprehensive predictor set. This pattern is consistent with the broader literature on predictors of posttraumatic stress, where peritraumatic and posttraumatic risk factors typically overshadow pretraumatic characteristics (Brewin, Andrews, & Valentine, 2000). This finding emphasizes the necessity to evaluate the particular stressors of the pandemic and their pandemic-related impacts.

Previous analyses on the effects of the timing of predictors using mixed-effects models indicated significant time interactions for few variables including a prior mental disorder, trauma exposure, income loss, and crisis management burden (Lotzin et al., 2024). In the context of our MERF, the timepoint during the pandemic and its interaction with other predictors demonstrated minimal importance (ranked >100; interaction strengths H < 0.5 on a 0–100 scale), suggesting that associations between stressors and AjD symptoms remained relatively stable across pandemic phases. In the context of random forests, variables exhibiting the greatest importance, along with their inherent interactions via tree splits, have been shown to prevail over minor time-dependent fluctuations. While it is acknowledged that linear (mixed) models have the capacity to detect statistically significant changes over time, the results of this study suggest that such effects contribute only marginally to overall predictive accuracy once dominant pandemic stressors are taken into consideration. The levels of AjD symptoms also remained relatively stable across the three measurement points, with only minor fluctuations over time. This stability indicates that, at the population level, distress related to the pandemic persisted rather than demonstrating clear remission or escalation.

It seems important to note that the streamlined MERF model comprising the top seven predictors achieved explanatory power close to that of a full model with over 240 predictors. This degree of parsimony is challenging to attain using conventional regression techniques, which frequently encounter difficulties with high-dimensional data, multicollinearity, and complex interactions. Random forests have been shown to more effectively capture non-linear relationships and interactions among predictors, thus enabling the identification of a subset of variables that retains high predictive accuracy.

The effect sizes of the seven most significant predictors derived from MERF were estimated using a mixed-effects linear regression model. The findings of this model were then compared with the reduced MERF model including the same seven predictors. The linear mixed-effects model with these predictors achieved a very good model fit, even without including interactions or non-linear relationships, which was similar to the model fit of the MERF. The findings indicate that the random forest method is well suited to the selection of a subset of predictors based on their importance estimates. These estimates can then be analyzed in a linear model to obtain interpretable effect estimates for individual predictors.

### Clinical and public health implications

Our findings have actionable implications for mental health interventions in ongoing and future public health crises. Targeted screening for AjD could prioritize individuals reporting high levels of uncertainty and current poor health, followed by assessments of social isolation, home conflicts, and routine disruption. Interventions that enhance a sense of control through clear communication, help individuals re-establish daily routines, and strengthen social connectedness may be particularly effective in mitigating AjD symptoms during future public health crises. Interventions that enhance tolerance of uncertainty (e.g. uncertainty management training) may also mitigate maladaptive adjustment trajectories.

Public health communications should aim to reduce ambiguous messaging around crisis duration and risk, and support services should include psychoeducation on coping with disruption to daily life while also fostering a sense of control and agency in individuals.

### Strengths and limitations

Strengths of this study include its large, diverse sample across 11 countries, the longitudinal design spanning three pandemic phases, and the comprehensive assessment of pandemic specific stressors. The use of MERF enabled the integration of complex, nonlinear interactions without a priori specification, and the incorporation of random effects accounted for intraindividual dependencies. However, several limitations merit consideration. First, reliance on self-report measures may introduce bias. Second, while random forests excel at prediction, they do not provide effect size estimates or directional inferences, necessitating complementary analyses to elucidate potential causal pathways. There are also no established cut-off criteria for selecting the most important predictors. Therefore, our decision to select exactly seven predictors based on visual inspection could be seen as subjective. Third, attrition across waves may limit generalizability to those experiencing the most severe distress. Finally, we did not include the occurrence of a current mental disorder as a predictor, as its inclusion could entail substantial symptom overlap with the dependent variable, risking criterion contamination and overadjustment. Consequently, we were unable to assess whether other current mental disorders constitute an independent predictor of adjustment disorder.

Further research should validate the identified most important predictors in general population or clinical samples and examine whether targeted interventions on the identified stressors effectively reduce AjD symptoms. Longitudinal mediation analyses could elucidate mechanisms linking uncertainty and health perceptions to AjD onset. Exploring additional protective factors beyond resilience and adaptive coping by using machine learning frameworks may identify buffers against pandemic related distress. Finally, integrating objective health data and ecological momentary assessments could refine the temporal dynamics of stress exposure and symptom fluctuations.

Our findings, based on a novel machine learning approach combining mixed model and forest analysis, highlight the pre-eminence of pandemic specific stressors, particularly uncertainty and perceived health vulnerability, in predicting AjD symptoms. By ranking the relative importance of a wide array of predictors, this study provides a data-driven basis for focused screening and tailored interventions, thereby informing evidence-based strategies to strengthen mental health resilience during prolonged public health emergencies.

Our findings also illustrates the potential of machine learning methods, which go beyond the assumptions of classical regression by modeling complex, non-linear relationships and interactions. This methodological advantage, combined with their capacity to handle high-dimensional data, may make machine learning approaches particularly powerful for improving prediction accuracy in mental health research.

## Supporting information

10.1017/S0033291726104048.sm001 Lotzin et al. supplementary material Lotzin et al. supplementary material

## Data Availability

The anonymized data of the ADJUST study are deposited in a repository (osf.io/js7ne) and can be accessed by researchers upon reasonable request via adjust@uke.de.

## Supplementary material

The supplementary material for this article can be found at http://doi.org/10.1017/S0033291726104048.
