# Supplementary material for: Determining the relative importance of risk and protective factors for adjustment disorder symptoms during the COVID-19 pandemic by mixed-effects random forests
Source: Psychol Med. 2026 Jun 4;56:e181. doi: 10.1017/S0033291726104048 (PMC13247793; doi:10.1017/S0033291726104048)
Supplement: Lotzin et al. supplementary material [file S0033291726104048sup001.docx]

**Supplement 1**

*Association, Importance and Interaction Strength of the 245 Predictors for Adjustment Disorder Symptoms*

| No. | Domain (a) | Item | Type | Assoc. with AjD (b) | Permuta-tion im-portance | Rank | Category | Mean impor-tance | Interaction strength(c) | *M* | *SD* | *N* | % |
| --- | --- | --- | --- | --- | --- | --- | --- | --- | --- | --- | --- | --- | --- |
| 1 | Fear of infection | Uncertainty about duration and risks of the pandemic | num | **+** | 2.773 | 2 | Important | 32.824 | 16.008 | 1.886 | 0.922 |  |  |
| 2 | Current situation | Current (poor) health | num | **+** | 1.968 | 1 | Important | 33.719 | 13.181 | 2.126 | 0.883 |  |  |
| 3 | Restricted personal contact | Social isolation | num | **+** | 1.357 | 6 | Important | 24.621 | 17.764 | 1.415 | 1.054 |  |  |
| 4 | Difficult housing condition | Conflicts at home | num | **+** | 0.810 | 4 | Important | 26.456 | 12.497 | 0.534 | 0.873 |  |  |
| 5 | Restricted activity | Loss of daily structure | num | **+** | 0.786 | 5 | Important | 24.644 | 9.921 | 1.211 | 1.106 |  |  |
| 6 | Fear of infection | Fear of getting infected with the coronavirus | num | **+** | 0.630 | 3 | Important | 26.494 | 6.120 | 1.414 | 0.984 |  |  |
| 7 | Restricted personal contact | Restricted personal contact with loved ones | num | **+** | 0.523 | 13 | Important | 20.193 | 4.486 | 1.661 | 1.056 |  |  |
| 8 | Restricted activity | Being at home most of the time | num | **+** | 0.403 | 8 | Important | 21.513 | 5.016 | 1.377 | 1.082 |  |  |
| 9 | Restricted personal contact | Restricted physical closeness to loved ones | num | **+** | 0.370 | 7 | Important | 21.638 | 5.711 | 1.508 | 1.116 |  |  |
| 10 | Difficult housing condition | No place of retreat | num | **+** | 0.336 | 10 | Important | 20.953 | 3.075 | 0.642 | 0.993 |  |  |
| 11 | Restricted personal contact | Restricted personal contact with others | num | **+** | 0.318 | 16 | Important | 19.023 | 3.414 | 1.510 | 1.016 |  |  |
| 12 | Fear of infection | Fear that loved ones get infected | num | **+** | 0.307 | 11 | Important | 20.337 | 4.042 | 1.853 | 1.031 |  |  |
| 13 | Work-related problems | (Threat of) job loss | num | **+** | 0.276 | 14 | Important | 20.184 | 4.889 | 0.524 | 0.981 |  |  |
| 14 | Work-related problems | (Threat of) income loss | num | **+** | 0.255 | 12 | Important | 20.290 | 3.421 | 0.685 | 1.067 |  |  |
| 15 | Work-related problems | Insufficient financial support by government | num | **+** | 0.227 | 19 | Important | 18.214 | 6.118 | 0.520 | 0.981 |  |  |
| 16 | Difficult housing condition | Restricted housing conditions | num | **+** | 0.223 | 18 | Important | 18.630 | 2.279 | 0.638 | 0.964 |  |  |
| 17 | Resilience (T1) | I have confidence in myself | num | **-** | 0.204 | 26 | Important | 17.126 | 3.396 | 2.852 | 0.920 |  |  |
| 18 | Resilience (T1) | I believe in myself | num | **-** | 0.196 | 21 | Important | 17.918 | 2.933 | 2.850 | 0.969 |  |  |
| 19 | Work-related problems | Increased workload | num | **+** | 0.195 | 9 | Important | 21.041 | 2.681 | 0.892 | 1.123 |  |  |
| 20 | Resilience (T1) | I am resilient | num | **-** | 0.175 | 20 | Important | 18.169 | 3.192 | 2.726 | 0.956 |  |  |
| 21 | Work-related problems | Working in close contact with people who could be infected | num | **+** | 0.172 | 15 | Important | 19.318 | 1.559 | 0.755 | 1.043 |  |  |
| 22 | Restricted activity | Restricted everyday activity | num | **+** | 0.165 | 22 | Important | 17.737 | 4.003 | 1.332 | 0.990 |  |  |
| 23 | Restricted personal contact | Unable to visit loved ones in a critical health condition | num | **+** | 0.164 | 30 | Important | 16.344 | 1.394 | 0.831 | 1.156 |  |  |
| 24 | Previous diagnosis mental disorder | Never had mental disorder | binary | **-** | 0.162 | 24 | Important | 17.454 | 2.013 |  |  | 21203 | 77.26 |
| 25 | Work-related problems | Not being able to work | num | **+** | 0.144 | 28 | Important | 16.455 | 2.351 | 0.461 | 0.938 |  |  |
| 26 | Crisis management and communication | Media coverage of the coronavirus pandemic | num | **+** | 0.142 | 25 | Important | 17.389 | 3.726 | 1.345 | 1.074 |  |  |
| 27 | Resilience (T1) | I appreciate myself | num | **-** | 0.130 | 29 | Important | 16.379 | 1.919 | 2.715 | 0.996 |  |  |
| 28 | Resilience (T1) | I can easily adjust in a difficult situation. | num | **-** | 0.129 | 27 | Important | 16.772 | 2.444 | 2.789 | 0.921 |  |  |
| 29 | Resilience (T1) | I can cope well with unexpected problems | num | **-** | 0.125 | 32 | Important | 15.770 | 1.940 | 2.688 | 0.938 |  |  |
| 30 | Socio-demographics | Age | num | **-** | 0.122 | 54 | Important | 12.101 | 1.803 | 44.358 | 15.322 |  |  |
| 31 |  | Other pandemic-specific burden | num | **+** | 0.117 | 17 | Important | 18.918 | 1.188 | 0.468 | 0.988 |  |  |
| 32 | Restricted activity | Restricted physical activity | num | **+** | 0.104 | 45 | Important | 13.212 | 1.188 | 1.309 | 1.099 |  |  |
| 33 | PCS: Joyful Activities | I have been doing something that I enjoy | num | **-** | 0.103 | 35 | Important | 14.716 | 1.329 | 1.955 | 0.847 |  |  |
| 34 | Burden of infection | Infection of loved ones with the coronavirus | num | **+** | 0.101 | 48 | Important | 12.635 | 0.956 | 0.858 | 1.123 |  |  |
| 35 | Fear of infection | Fear of infecting others with the coronavirus | num | **+** | 0.098 | 38 | Important | 14.240 | 2.376 | 1.354 | 1.130 |  |  |
| 36 | Crisis management and communication | Poor information from the government | num | **+** | 0.090 | 31 | Important | 16.013 | 1.075 | 1.157 | 1.042 |  |  |
| 37 | Resilience (T1) | I can easily pick up where I left off | num | **-** | 0.088 | 33 | Important | 15.132 | 1.113 | 2.719 | 0.926 |  |  |
| 38 | Current situation | Amount reduction of household income due to pandemic (Euro) | num | **+** | 0.087 | 49 | Important | 12.621 | 1.311 | 223.514 | 990.486 |  |  |
| 39 | Current service use | None of those services | binary | **-** | 0.082 | 39 | Important | 14.209 | 1.185 |  |  | 21654 | 78.91 |
| 40 | Crisis management and communication | Poor crisis management of the government | num | **+** | 0.082 | 41 | Important | 13.863 | 1.497 | 1.312 | 1.111 |  |  |
| 41 | Current situation | Weeks spent more time at home | num | **+** | 0.081 | 62 | Important | 9.876 | 2.011 | 13.640 | 15.159 |  |  |
| 42 | Restricted activity | Restricted leisure activity | num | **+** | 0.077 | 53 | Important | 12.249 | 0.879 | 1.644 | 1.055 |  |  |
| 43 | Work-related problems | Reduced working hours | num | **+** | 0.075 | 37 | Important | 14.265 | 1.336 | 0.473 | 0.925 |  |  |
| 44 | Resilience (T1) | I can handle a lot at same time | num | **-** | 0.075 | 42 | Important | 13.503 | 2.375 | 2.618 | 1.013 |  |  |
| 45 | Work-related problems | Other work-related burden | num | **+** | 0.072 | 23 | Important | 17.585 | 1.152 | 0.131 | 0.549 |  |  |
| 46 | Restricted activity | Other burden due to restricted public life | num | **+** | 0.071 | 34 | Important | 14.740 | 0.716 | 0.211 | 0.686 |  |  |
| 47 | Restricted personal contact | Unable to attend the funeral of loved ones | num | **+** | 0.071 | 50 | Important | 12.484 | 1.605 | 0.496 | 0.978 |  |  |
| 48 | Work-related problems | Working from home (home office) | num | **+** | 0.069 | 43 | Important | 13.398 | 0.833 | 0.713 | 1.012 |  |  |
| 49 | Restricted access to resources | Insufficient capacity of health system | num | **+** | 0.065 | 58 | Important | 10.995 | 1.266 | 1.122 | 1.130 |  |  |
| 50 | Lockdown stringency | Stringency of pandemic measures | num | **+** | 0.065 | 87 | Important | 7.609 | 2.204 | 60.360 | 11.463 |  |  |
| 51 | Socio-demographics | Working hours | num | **-** | 0.064 | 73 | Important | 8.707 | 3.115 | 29.647 | 16.534 |  |  |
| 52 | Restricted activity | Restricted private travel | num | **+** | 0.061 | 65 | Important | 9.513 | 1.396 | 1.689 | 1.106 |  |  |
| 53 | Burden of infection | My own infection with the coronavirus | num | **+** | 0.060 | 46 | Important | 12.748 | 0.675 | 0.559 | 0.916 |  |  |
| 54 | Difficult housing conditions | Violent assaults at home | num | **+** | 0.060 | 55 | Important | 11.884 | 0.832 | 0.089 | 0.424 |  |  |
| 55 | Burden of infection | Being rejected because of infection | num | **+** | 0.060 | 44 | Important | 13.303 | 0.866 | 0.342 | 0.765 |  |  |
| 56 | Restricted access to resources | Restricted access to regular health care/medication | num | **+** | 0.060 | 57 | Important | 11.503 | 0.578 | 0.836 | 1.022 |  |  |
| 57 | Socio-demographics | Sex: Male | binary | **-** | 0.058 | 56 | Important | 11.528 | 1.307 |  |  | 8598 | 31.33 |
| 58 | Burden of infection | Death of a loved one due to the coronavirus infection | num | **+** | 0.058 | 59 | Important | 10.574 | 0.830 | 0.634 | 1.093 |  |  |
| 59 | Restricted personal contact | Other burden due to restricted social contact | num | **+** | 0.054 | 36 | Important | 14.643 | 0.457 | 0.141 | 0.563 |  |  |
| 60 | Restricted access to resources | Restricted access to goods | num | **+** | 0.050 | 69 | Important | 9.162 | 0.637 | 0.484 | 0.806 |  |  |
| 61 | Positive consequences | Time to recover from normal daily stress | num | **-** | 0.045 | 86 | Important | 7.624 | 1.271 | 1.363 | 1.162 |  |  |
| 62 | Positive consequences | More quality time with loved ones, friends, or pet | num | **-** | 0.045 | 82 | Important | 7.838 | 1.059 | 1.971 | 1.039 |  |  |
| 63 | Resilience (T1) | I am able to persevere | num | **-** | 0.044 | 63 | Important | 9.857 | 0.795 | 2.983 | 0.861 |  |  |
| 64 | Burden of infection | Having infected others with the coronavirus | num | **+** | 0.043 | 60 | Important | 10.315 | 0.874 | 0.612 | 0.984 |  |  |
| 65 | PCS: Prevention Adherence | Informing myself about the current state of the pandemic | num | **+** | 0.042 | 66 | Important | 9.501 | 1.117 | 2.327 | 0.833 |  |  |
| 66 | ACE questionnaire | Emotional neglect | binary | **+** | 0.041 | 52 | Important | 12.413 | 0.544 |  |  | 5212 | 18.99 |
| 67 | Difficult housing condition | Other burden at home | num | **+** | 0.039 | 47 | Important | 12.741 | 0.657 | 0.108 | 0.495 |  |  |
| 68 | Restricted activity | Restricted religious or spiritual activities | num | **+** | 0.038 | 72 | Important | 8.859 | 0.708 | 0.521 | 0.932 |  |  |
| 69 | Positive consequences | More time for enjoyable activities | num | **-** | 0.038 | 91 | Important | 7.176 | 0.902 | 1.348 | 1.157 |  |  |
| 70 | Work-related problems | Restricted work travel | num | **+** | 0.036 | 81 | Important | 7.937 | 0.510 | 0.486 | 0.905 |  |  |
| 71 | Traumatic experience (PCL-5) during pandemic | Involved actual or threatened death, serious injury, or sexual violence | binary | **+** | 0.036 | 51 | Important | 12.448 | 0.365 |  |  | 1615 | 5.88 |
| 72 | Fear of infection | Fear of infecting others with the coronavirus | num | **+** | 0.036 | 40 | Important | 14.181 | 0.249 | 0.158 | 0.593 |  |  |
| 73 | Current service use | Online courses (e.g. stress management) | binary | **+** | 0.035 | 80 | Important | 8.025 | 1.034 |  |  | 1358 | 4.95 |
| 74 | PCS: Healthy Lifestyle | I have been relaxing | num | **+** | 0.035 | 79 | Important | 8.067 | 1.798 | 1.109 | 1.037 |  |  |
| 75 | Current situation | Perceived risk for severe symptoms of coronavirus disease | binary | **+** | 0.033 | 61 | Important | 10.173 | 0.738 |  |  | 6719 | 24.48 |
| 76 | Living situation | Number of close friends | num | **-** | 0.031 | 93 | Important | 6.879 | 1.591 | 4.958 | 4.528 |  |  |
| 77 | Current situation | Hours per day outside home | num | **-** | 0.029 | 127 | Important | 4.125 | 1.274 | 4.635 | 4.211 |  |  |
| 78 | Positive consequences | Learning of new skills to solve problems | num | **-** | 0.028 | 99 | Important | 5.933 | 1.092 | 1.508 | 1.116 |  |  |
| 79 | Socio-demographics | Reduced monthly household income | binary | **+** | 0.028 | 71 | Important | 9.050 | 0.798 |  |  | 7766 | 28.30 |
| 80 | Positive consequences | Cohesion in society | num | **-** | 0.028 | 97 | Important | 6.337 | 1.257 | 1.590 | 0.996 |  |  |
| 81 | Positive consequences | Learning new communication technologies | num | **-** | 0.027 | 110 | Important | 5.210 | 1.648 | 1.708 | 1.153 |  |  |
| 82 | Problems with childcare | Loss of childcare | num | **+** | 0.025 | 77 | Important | 8.116 | 0.087 | 0.303 | 0.794 |  |  |
| 83 | Country | Poland | binary | **+** | 0.025 | 92 | Important | 6.971 | 0.339 |  |  | 4388 | 15.99 |
| 84 | PCS: Healthy Lifestyle | I have been exercising or taking a walk | num | **-** | 0.023 | 138 | Important | 3.715 | 0.838 | 1.803 | 0.970 |  |  |
| 85 | Helpful services | None of those services | binary | **-** | 0.023 | 85 | Important | 7.777 | 0.730 |  |  | 9633 | 35.10 |
| 86 | ACE questionnaire | Sexual abuse | binary | **+** | 0.023 | 83 | Important | 7.832 | 0.361 |  |  | 2428 | 8.85 |
| 87 | Problems with childcare | Difficulties with combining work with childcare | num | **+** | 0.023 | 74 | Important | 8.606 | 0.578 | 0.383 | 0.874 |  |  |
| 88 | Positive consequences | Appreciation of own health or health of loved ones | num | **-** | 0.022 | 94 | Important | 6.627 | 0.917 | 2.252 | 0.910 |  |  |
| 89 | PCS: Daily Structure | I have been structuring my day | num | **-** | 0.022 | 125 | Important | 4.200 | 0.839 | 1.765 | 0.957 |  |  |
| 90 | Current service use | Other service currently used | binary | **+** | 0.022 | 90 | Important | 7.390 | 0.545 |  |  | 5293 | 19.29 |
| 91 | Positive consequences | Working from home | num | **-** | 0.021 | 131 | Important | 4.078 | 0.615 | 1.256 | 1.211 |  |  |
| 92 | PCS: Healthy Lifestyle | I have been paying attention to a healthy diet | num | **+** | 0.021 | 124 | Important | 4.218 | 0.463 | 1.906 | 0.928 |  |  |
| 93 | PCS: Joyful Activities | I have been spending a good time with loved ones, friends, or my pet | num | **-** | 0.021 | 106 | Important | 5.397 | 0.962 | 1.878 | 0.958 |  |  |
| 94 | Positive consequences | Time to rethink priorities in life | num | **-** | 0.020 | 119 | Important | 4.547 | 0.713 | 1.727 | 1.110 |  |  |
| 95 | Current situation | Recovered from mental disorder | binary | **+** | 0.020 | 84 | Important | 7.822 | 0.411 |  |  | 3875 | 14.12 |
| 96 | Living situation | Number of people living together | num | **-** | 0.020 | 118 | Important | 4.554 | 1.148 | 1.519 | 1.798 |  |  |
| 97 | Current service use | Personal psychotherapy | binary | **+** | 0.020 | 70 | Important | 9.113 | 0.428 |  |  | 1842 | 6.71 |
| 98 | Positive consequences | New job opportunity or more work orders | num | **-** | 0.020 | 133 | Important | 3.977 | 0.391 | 0.759 | 1.045 |  |  |
| 99 | ACE questionnaire | Psychological abuse | binary | **+** | 0.020 | 68 | Important | 9.164 | 0.481 |  |  | 4711 | 17.17 |
| 100 | PCS: Daily Structure | I have been paying attention to maintain my daily routine | num | **-** | 0.020 | 126 | Important | 4.152 | 0.888 | 1.874 | 0.932 |  |  |
| 101 | PCS: Joyful Activities | I have been doing something useful, e.g. tidying up or cleaning | num | **+** | 0.020 | 108 | Important | 5.223 | 1.287 | 1.829 | 0.908 |  |  |
| 102 | Country | Portugal | binary | **+** | 0.019 | 113 | Important | 4.975 | 0.191 |  |  | 1077 | 3.92 |
| 103 | PCS: Joyful Activities | Spending a good time with loved ones/ friends through digital media | num | **+** | 0.018 | 134 | Important | 3.931 | 0.861 | 1.798 | 0.903 |  |  |
| 104 | Current situation | Severity of coronavirus infection of known person | num | **+** | 0.018 | 103 | Important | 5.570 | 0.852 | 0.499 | 1.010 |  |  |
| 105 | Country | Germany | binary | **+** | 0.018 | 75 | Important | 8.559 | 0.634 |  |  | 4406 | 16.06 |
| 106 | Traumatic experience (PCL-5) during pandemic | Happened to a close family member or friend | binary | **+** | 0.018 | 67 | Important | 9.326 | 0.339 |  |  | 1107 | 4.03 |
| 107 | Country | Netherlands | binary | **-** | 0.017 | 89 | Important | 7.471 | 0.307 |  |  | 3347 | 12.20 |
| 108 | PCS: Healthy Lifestyle | I have been paying attention to good sleeping habits | num | **-** | 0.016 | 98 | Important | 5.959 | 0.645 | 1.825 | 0.960 |  |  |
| 109 | PCS: Healthy Lifestyle | I have been paying attention to take enough breaks | num | **-** | 0.016 | 141 | Undecided | 3.407 | 0.760 | 1.516 | 0.984 |  |  |
| 110 | Helpful services | Personal psychotherapy | binary | **+** | 0.016 | 115 | Important | 4.854 | 0.817 |  |  | 9685 | 35.29 |
| 111 | PCS: Prevention Adherence | Following the recommendations to limit spread of coronavirus | num | **+** | 0.015 | 109 | Important | 5.213 | 0.439 | 2.649 | 0.658 |  |  |
| 112 | Positive consequences | Less working hours | num | **-** | 0.015 | 145 | Undecided | 3.243 | 0.515 | 0.673 | 1.024 |  |  |
| 113 | Positive consequences | (Potentially) increased income | num | **-** | 0.015 | 123 | Important | 4.267 | 0.757 | 0.519 | 0.938 |  |  |
| 114 | ACE questionnaire | Household dysfunction | binary | **+** | 0.015 | 78 | Important | 8.070 | 0.059 |  |  | 1061 | 3.87 |
| 115 | Traumatic experience (PCL-5) during pandemic | Direct experience | binary | **+** | 0.015 | 76 | Important | 8.474 | 0.469 |  |  | 1139 | 4.15 |
| 116 | Positive consequences | Appreciation of quality of healthcare in my country | num | **-** | 0.015 | 136 | Important | 3.850 | 0.628 | 1.996 | 1.052 |  |  |
| 117 | Current situation | More time at home: No | binary | **-** | 0.014 | 120 | Important | 4.498 | 0.408 |  |  | 7840 | 28.57 |
| 118 | Country | Croatia | binary | **-** | 0.014 | 64 | Important | 9.852 | 0.131 |  |  | 3187 | 11.61 |
| 119 | Traumatic experience (PCL-5) before pandemic | Involved actual or threatened death, serious injury, or sexual violence | binary | **+** | 0.014 | 105 | Important | 5.490 | 0.542 |  |  | 6081 | 22.16 |
| 120 | Socio-demographics | Retired | binary | **-** | 0.013 | 104 | Important | 5.565 | 0.126 |  |  | 3565 | 12.99 |
| 121 | Traumatic experience (PCL-5) before pandemic | Direct experience | binary | **+** | 0.013 | 101 | Important | 5.898 | 0.289 |  |  | 4460 | 16.25 |
| 122 | Socio-demographics | Current financial support by government: Not answered | binary | **-** | 0.013 | 107 | Important | 5.336 | 0.672 |  |  | 7568 | 27.58 |
| 123 | Helpful services | Online courses (e.g., stress management) | binary | **+** | 0.011 | 148 | Undecided | 3.144 | 0.561 |  |  | 4084 | 14.88 |
| 124 | Socio-demographics | Work status: Full-time employment | binary | **-** | 0.011 | 114 | Important | 4.974 | 0.521 |  |  | 13706 | 49.94 |
| 125 | Socio-demographics | Work status: Part-time | binary | **+** | 0.011 | 111 | Important | 5.039 | 0.978 |  |  | 4233 | 15.42 |
| 126 | Socio-demographics | Employment area: Not working | binary | **+** | 0.010 | 121 | Important | 4.382 | 0.258 |  |  | 5881 | 21.43 |
| 127 | Current service use | Online psychotherapy | binary | **+** | 0.010 | 88 | Important | 7.578 | 0.163 |  |  | 709 | 2.58 |
| 128 | Helpful services | Online psychotherapy | binary | **+** | 0.010 | 132 | Important | 4.006 | 0.568 |  |  | 4384 | 15.97 |
| 129 | ACE questionnaire | Physical abuse | binary | **+** | 0.009 | 102 | Important | 5.646 | 0.733 |  |  | 2817 | 10.26 |
| 130 | Socio-demographics | Income: Very low | binary | **+** | 0.009 | 137 | Important | 3.831 | 0.876 |  |  | 1989 | 7.25 |
| 131 | Socio-demographics | Work status: Vocational training or study | binary | **+** | 0.009 | 130 | Important | 4.088 | 0.470 |  |  | 3737 | 13.62 |
| 132 | Current service use | Online coaching | binary | **+** | 0.009 | 117 | Important | 4.555 | 0.154 |  |  | 410 | 1.49 |
| 133 | Time point | T3 (versus T1) | binary | **-** | 0.008 | 188 | Unimportant | 1.076 | 0.316 |  |  | 5639 | 20.55 |
| 134 | Current service use | Telephone consultation | binary | **+** | 0.008 | 95 | Important | 6.466 | 0.045 |  |  | 862 | 3.14 |
| 135 | Socio-demographics | Number of children | num | **-** | 0.008 | 157 | Undecided | 2.832 | 0.688 | 1.135 | 1.203 |  |  |
| 136 | Time point | T2 (versus T1) | binary | **+** | 0.008 | 175 | Unimportant | 1.676 | 0.417 |  |  | 6716 | 24.47 |
| 137 | Socio-demographics | Employment area: Services (e.g. hairdresser, waitress) | binary | **+** | 0.008 | 164 | Undecided | 2.272 | 0.093 |  |  | 1786 | 6.51 |
| 138 | Socio-demographics | Current financial support by government: Yes | binary | **+** | 0.008 | 116 | Important | 4.626 | 0.578 |  |  | 1676 | 6.11 |
| 139 | Current situation | Pandemic news consumption: 30-60 min/day | binary | **+** | 0.008 | 165 | Unimportant | 2.234 | 0.475 |  |  | 6751 | 24.60 |
| 140 | Current situation | Pandemic news consumption: None | binary | **-** | 0.008 | 156 | Undecided | 2.883 | 0.182 |  |  | 2340 | 8.53 |
| 141 | Living situation | Relationship status: Single | binary | **+** | 0.007 | 140 | Important | 3.529 | 0.217 |  |  | 7774 | 28.33 |
| 142 | Current service use | Personal coaching | binary | **+** | 0.007 | 112 | Important | 5.010 | 0.143 |  |  | 623 | 2.27 |
| 143 | Current situation | Pandemic news consumption: 2-3 hours/day | binary | **+** | 0.007 | 122 | Important | 4.309 | 0.180 |  |  | 887 | 3.23 |
| 144 | Socio-demographics | Work status: Other | binary | **+** | 0.007 | 128 | Important | 4.117 | 0.322 |  |  | 1827 | 6.66 |
| 145 | Living situation | Living together with other people: Yes | binary | **-** | 0.006 | 152 | Undecided | 3.022 | 0.186 |  |  | 19164 | 69.83 |
| 146 | Socio-demographics | Employment area: Education (e.g. teacher, lecturer) | binary | **+** | 0.006 | 159 | Undecided | 2.736 | 0.542 |  |  | 3647 | 13.29 |
| 147 | Current situation | Pandemic news consumption: >=3 hours/day | binary | **+** | 0.006 | 100 | Important | 5.902 | 0.062 |  |  | 791 | 2.88 |
| 148 | Living situation | Relationship status: Temporary relationship | binary | **+** | 0.006 | 160 | Undecided | 2.713 | 0.034 |  |  | 861 | 3.14 |
| 149 | Helpful services | Personal coaching | binary | **+** | 0.006 | 147 | Undecided | 3.164 | 0.666 |  |  | 6519 | 23.75 |
| 150 | Living situation | Living with parents | binary | **+** | 0.006 | 155 | Undecided | 2.897 | 0.437 |  |  | 4312 | 15.71 |
| 151 | Socio-demographics | Daily physical contact at work | binary | **-** | 0.006 | 161 | Undecided | 2.538 | 0.420 |  |  | 17588 | 64.09 |
| 152 | Country | Georgia | binary | **+** | 0.006 | 96 | Important | 6.371 | 0.259 |  |  | 1173 | 4.27 |
| 153 | Country | Greece | binary | **+** | 0.005 | 142 | Important | 3.344 | 0.122 |  |  | 1082 | 3.94 |
| 154 | Living situation | Living with partner and children | binary | **-** | 0.005 | 162 | Undecided | 2.408 | 0.309 |  |  | 6649 | 24.23 |
| 155 | Current situation | Pandemic news consumption: 1-2 hours/day | binary | **+** | 0.005 | 144 | Important | 3.247 | 0.415 |  |  | 2361 | 8.60 |
| 156 | Socio-demographics | Employment area: Health care (e.g. nurse, care assistant, front-line health worker) | binary | **-** | 0.005 | 173 | Unimportant | 1.723 | 0.086 |  |  | 3658 | 13.33 |
| 157 | Socio-demographics | Household income: medium | binary | **+** | 0.005 | 186 | Unimportant | 1.119 | 0.298 |  |  | 9954 | 36.27 |
| 158 | Socio-demographics | Highest education: completed vocational studies | binary | **+** | 0.005 | 139 | Important | 3.547 | 0.225 |  |  | 4005 | 14.59 |
| 159 | Helpful services | Online self-help group | binary | **+** | 0.005 | 181 | Unimportant | 1.361 | 0.245 |  |  | 2237 | 8.15 |
| 160 | Living situation | Living with children only | binary | **+** | 0.005 | 149 | Undecided | 3.131 | 0.235 |  |  | 2110 | 7.69 |
| 161 | Socio-demographics | Work status: Seeking work | binary | **+** | 0.004 | 154 | Undecided | 2.918 | 0.317 |  |  | 1513 | 5.51 |
| 162 | Socio-demographics | Community: Rural | binary | **-** | 0.004 | 172 | Unimportant | 1.776 | 0.242 |  |  | 3858 | 14.06 |
| 163 | Traumatic experience (PCL-5) before pandemic | Happened to a close family member or friend | binary | **+** | 0.004 | 146 | Undecided | 3.230 | 0.774 |  |  | 3001 | 10.94 |
| 164 | Socio-demographics | Highest education: 10-13 years of schooling | binary | **+** | 0.004 | 174 | Unimportant | 1.720 | 0.923 |  |  | 6213 | 22.64 |
| 165 | Current service use | Online self-help group | binary | **+** | 0.004 | 135 | Important | 3.929 | 0.044 |  |  | 339 | 1.24 |
| 166 | Socio-demographics | Household income: low | binary | **+** | 0.004 | 179 | Unimportant | 1.430 | 0.292 |  |  | 5211 | 18.99 |
| 167 | Current situation | Personal contact with loved ones or friends: less than once/week | binary | **+** | 0.003 | 184 | Unimportant | 1.207 | 0.311 |  |  | 6429 | 23.43 |
| 168 | Living situation | Having children: Yes | binary | **-** | 0.003 | 163 | Undecided | 2.281 | 0.327 |  |  | 15721 | 57.29 |
| 169 | Socio-demographics | Parents born in another country | binary | **+** | 0.003 | 158 | Undecided | 2.827 | 0.624 |  |  | 7454 | 27.16 |
| 170 | Current situation | Personal contact with loved ones or friends: No contact | binary | **+** | 0.003 | 150 | Undecided | 3.099 | 0.450 |  |  | 2309 | 8.41 |
| 171 | Helpful services | Personal self-help group | binary | **+** | 0.003 | 176 | Unimportant | 1.661 | 0.292 |  |  | 3444 | 12.55 |
| 172 | Living situation | Having close friends | binary | **-** | 0.003 | 151 | Important | 3.065 | 0.366 |  |  | 24593 | 89.61 |
| 173 | Current service use | Personal self-help group | binary | **+** | 0.003 | 143 | Undecided | 3.258 | 0.025 |  |  | 254 | 0.93 |
| 174 | Living situation | Living with partner | binary | **-** | 0.002 | 190 | Unimportant | 0.919 | 0.483 |  |  | 6936 | 25.27 |
| 175 | Country | Austria | binary | **-** | 0.002 | 170 | Unimportant | 1.829 | 0.210 |  |  | 1617 | 5.89 |
| 176 | Helpful services | Telephone consultation | binary | **+** | 0.002 | 153 | Undecided | 2.972 | 0.359 |  |  | 3695 | 13.46 |
| 177 | Current situation | Severity of infection: Moderate | binary | **+** | 0.002 | 168 | Unimportant | 1.886 | 0.077 |  |  | 689 | 2.51 |
| 178 | Socio-demographics | Work status: Freelancer | binary | **+** | 0.002 | 167 | Unimportant | 2.020 | 0.202 |  |  | 1064 | 3.88 |
| 179 | Current situation | More time at home? - Yes. because of quarantine | binary | **+** | 0.002 | 193 | Unimportant | 0.823 | 0.018 |  |  | 660 | 2.40 |
| 180 | Country | Lithuania | binary | **+** | 0.002 | 129 | Important | 4.091 | 0.865 |  |  | 1054 | 3.84 |
| 181 | Helpful services | Online Coaching | binary | **+** | 0.001 | 178 | Unimportant | 1.440 | 0.324 |  |  | 3321 | 12.10 |
| 182 | Current situation | Personal contact with loved ones or friends: Once/week | binary | **+** | 0.001 | 204 | Unimportant | 0.557 | 0.315 |  |  | 3946 | 14.38 |
| 183 | Current situation | Virtual contact with loved ones: Less than once/week | binary | **+** | 0.001 | 200 | Unimportant | 0.678 | 0.163 |  |  | 2952 | 10.76 |
| 184 | Traumatic experience (PCL-5) before pandemic | Exposed as part of job | binary | **-** | 0.001 | 199 | Unimportant | 0.688 | 0.387 |  |  | 675 | 2.46 |
| 185 | Socio-demographics | Work status: Self-employed | binary | **-** | 0.001 | 183 | Unimportant | 1.248 | 0.056 |  |  | 1632 | 5.95 |
| 186 | Living situation | Living with colleagues/fellow students | binary | **+** | 0.001 | 201 | Unimportant | 0.659 | 2.696 |  |  | 929 | 3.39 |
| 187 | Socio-demographics | Highest education: < 10 years of schooling | binary | **-** | 0.001 | 208 | Unimportant | 0.329 | 0.030 |  |  | 637 | 2.32 |
| 188 | Country | Italy | binary | 0 | 0.001 | 182 | Unimportant | 1.261 | 0.308 |  |  | 451 | 1.64 |
| 189 | Socio-demographics | Refugee status | binary | **+** | 0.001 | 185 | Unimportant | 1.206 | 0.139 |  |  | 968 | 3.53 |
| 190 | Nationality | Finland | binary | **-** | 0.001 | 243 | Unimportant | -0.327 | 0.004 |  |  | 136 | 0.50 |
| 191 | Socio-demographics | Employment area: Public security (e.g. police, fire department) | binary | **-** | 0.000 | 166 | Unimportant | 2.205 | 0.024 |  |  | 1099 | 4.00 |
| 192 | Traumatic experience (PCL-5) during pandemic | Exposed as part of job | binary | **+** | 0.000 | 171 | Unimportant | 1.800 | 0.009 |  |  | 284 | 1.03 |
| 193 | Current situation | Infection treated in hospital | binary | **+** | 0.000 | 177 | Unimportant | 1.625 | 0.001 |  |  | 101 | 0.37 |
| 194 | Nationality | Other | binary | **+** | 0.000 | 191 | Unimportant | 0.857 | 0.005 |  |  | 130 | 0.47 |
| 195 | Current situation | Severity of infection: Severe (vs. No infection) | binary | **+** | 0.000 | 169 | Unimportant | 1.859 | 0.002 |  |  | 123 | 0.45 |
| 196 | Current situation | More time at home? - Yes. because of infection | binary | **+** | 0.000 | 213 | Unimportant | 0.188 | 0.017 |  |  | 561 | 2.04 |
| 197 | Socio-demographics | Sex: Other (vs. Female) | binary | **+** | 0.000 | 189 | Unimportant | 1.007 | 0.002 |  |  | 110 | 0.40 |
| 198 | Nationality | Thailand | binary | **+** | 0.000 | 205 | Unimportant | 0.523 | 0.000 |  |  | 35 | 0.13 |
| 199 | Current situation | Infection of someone known personally | binary | **+** | 0.000 | 241 | Unimportant | -0.219 | 0.001 |  |  | 37 | 0.13 |
| 200-234, 236, 237(d) | Nationality | Country nationalities | binary |  | 0.000 | 195-  245 | Unimportant | 0.000-1.375 |  |  |  | 1-57 | 0.001-0.21 |
| 235 | Current situation | Personal contact with loved ones or friends: 1-2 /week | binary | **-** | 0.000 | 202 | Unimportant | 0.605 | 0.275 |  |  | 5665 | 20.64 |
| 238 | Current situation | Virtual contact with loved ones or friends: 1-2 /week | binary | **-** | 0.000 | 214 | Unimportant | 0.172 | 0.351 |  |  | 5291 | 19.28 |
| 239 | Current situation | Severity of infection: mild (vs. No infection) | binary | **-** | 0.000 | 222 | Unimportant | 0.032 | 0.230 |  |  | 801 | 2.92 |
| 240 | Current situation | Virtual contact with loved ones or friends: No contact | binary | **-** | 0.000 | 215 | Unimportant | 0.159 | 0.993 |  |  | 883 | 3.22 |
| 241 | Socio-demographics | Community: Living in a suburb | binary | **-** | -0.001 | 187 | Unimportant | 1.108 | 0.179 |  |  | 2698 | 9.83 |
| 242 | Living situation | Relationship status: Stable living separately | binary | **+** | -0.001 | 207 | Unimportant | 0.394 | 0.561 |  |  | 2846 | 10.37 |
| 243 | Socio-demographics | Employment area: Maintenance. repair. construction (e.g.. cleaner. craftsmen) | binary | **-** | -0.001 | 192 | Unimportant | 0.837 | 0.112 |  |  | 866 | 3.16 |
| 244 | Socio-demographics | Community: Living in a small city | binary | **-** | -0.002 | 194 | Unimportant | 0.814 | 0.446 |  |  | 6733 | 24.53 |
| 245 | Current situation | Virtual contact with loved ones or friends: Once /week | binary | **-** | -0.003 | 242 | Unimportant | -0.308 | 0.246 |  |  | 2725 | 9.93 |

*Note.* ^(a)^ Domain: Questionnaire domain; name of subscale is given when item is part of a validated questionnaire. ^(b)^ Direction of association (regression within mixed-effects model) with levels of adjustment disorder (AjD) symptoms: + = positive association; − = negative association. The effect can be in the opposite direction in some splits of the data or at lower nodes within a tree. (c) Friedman’s H; transformed to range 0–100 for better readability. (d) Rank 200–234 and 236–237 not shown: individual nationalities with partly small case numbers, all with importance values of 0.0. PCS: Pandemic Coping Scale (Lotzin et al., 2022c). ACE questionnaire: Adverse Childhood Experiences Questionnaire (Felitti et al., 1998). PCL-5: Criterion A items of the PTSD Checklist for DSM-5 (Weathers et al., 2013).
